# Neurogenic pulmonary edema following febrile status epilepticus in a 22-month-old infant with multiple respiratory virus co-detection: a case report

**DOI:** 10.1186/s12879-020-05115-2

**Published:** 2020-06-01

**Authors:** Yoshie Takagi, Takeaki Imamura, Shota Endo, Kenta Hayashi, Satoka Akiyama, Yoji Ikuta, Takahiro Kawaguchi, Tomoko Sumita, Tatsuo Katori, Masanori Hashino, Shinji Saito, Takato Odagiri, Kunihiro Oba, Makoto Kuroda, Tsutomu Kageyama

**Affiliations:** 1 Department of Pediatrics, Showa General Hospital, 8-1-1 Hanakoganei, Kodaira, Tokyo, 187-8510 Japan; 2 Department of Emergency Medicine and Critical Care, Showa General Hospital, 8-1-1 Hanakoganei, Kodaira, Tokyo, 187-8510 Japan; 3 Department of Virology, Tohoku University Graduate School of Medicine, 2-1 Seiryo, Aoba, Sendai, 980-8575 Japan; 4 Pathogen Genomics Center, National Institute of Infectious Diseases, 1-23-1 Toyama, Shinjuku, Tokyo, 162-8640 Japan; 5 Influenza Virus Research Center, National Institute of Infectious Diseases, 4-7-1 Gakuen, Musashimurayama, Tokyo, 208-0011 Japan

**Keywords:** Neurogenic pulmonary edema, Febrile status epilepticus, Co-detection, Human coronavirus HKU1, Influenza C virus, Human parainfluenza virus 2, Multiplex real-time RT-PCR

## Abstract

**Background:**

Neurogenic pulmonary edema is a rare but serious complication of febrile status epilepticus in children. Comprehensive screening for viral pathogens is seldomly performed in the work-up of febrile children.

**Case presentation:**

A 22-month-old girl presented with her first episode of febrile status epilepticus, after which she developed acute pulmonary edema and respiratory failure. After the termination of seizure activity, the patient was intubated and managed on mechanical ventilation in the emergency room. The resolution of respiratory failure, as well as the neurological recovery, was achieved 9 h after admission, and the patient was discharged 6 days after admission without any complications. Molecular biological diagnostic methods identified the presence of human coronavirus HKU1, influenza C virus, and human parainfluenza virus 2 from the patient’s nasopharyngeal specimens.

**Conclusions:**

Neurogenic pulmonary edema following febrile status epilepticus was suspected to be the etiology of our patient’s acute pulmonary edema and respiratory failure. Timely seizure termination and rapid airway and respiratory intervention resulted in favorable outcomes of the patient. Molecular biological diagnostic methods identified three respiratory viruses; however, their relevance and association with clinical symptoms remain speculative.

## Background

Neurogenic pulmonary edema (NPE) is characterized by the development of pulmonary edema within minutes to hours of a significant central nervous system (CNS) insult, often resolving spontaneously within 24–48 h [1–3]. Febrile seizures (FS) is the most common convulsive event, affecting 2–5% of children younger than 60 months [4–7]. The majority of FS cases follow a benign clinical course; however, approximately 5–8% of FS cases develop status epilepticus (SE) [8, 9]. NPE following febrile SE in children is a rare complication, although its incidence rate might be underestimated [10–13].

Molecular biological diagnostic methods such as multiplex PCR and next generation sequencing (NGS) can detect pathogens from clinical specimens with high sensitivity; this also leads to co-detection of multiple pathogens from the same clinical specimen. With the increased application of multiplex PCR in clinical settings, there have been reports of multiple pathogen co-detection rates from 10 to 50% of human respiratory specimens [14–17]. It has also been revealed that multiple pathogens, both viral and bacterial, are prevalent even in respiratory specimens from healthy, asymptomatic children [14, 18]. It is sometimes difficult to interpret clinical relevance of detected pathogens by molecular biological diagnostic methods alone [19]. The interaction among co-detected multiple pathogens and their association with clinical symptoms remain controversial [17, 20–23].

We report the case of a 22-month-old girl with febrile SE who developed acute respiratory failure due to NPE. We also describe the co-detection of three respiratory viruses from the patient’s nasopharyngeal specimens using molecular biological diagnostic methods.

The Institutional Medical Ethics Committee of the National Institute of Infectious Diseases and the Ethics Committee of Showa General Hospital approved this study (Approval Nos. 553 and 677, and REC-094, respectively). The patient’s parents provided written informed consent for analysis of the patient’s clinical specimens and publication of this case report.

## Case presentation

The patient is a previously healthy 22-month-old girl without perinatal abnormalities or personal or family history of seizure. The patient had not been to any nursery school prior to the event. She had two siblings, 11 and 5 years old, who had no respiratory symptoms. Clonic seizure of the patient’s right side commenced 12 h after fever development, followed by generalized tonic and tonic-clonic seizures without self-resolution. The seizure activity had persisted for 40 min when the patient arrived at our emergency room (ER) by ambulance. Spontaneous breathing was absent with SpO_2_ of 50% upon ER arrival. Bag valve mask (BVM) ventilation supplied with 100% oxygen was commenced immediately, and SpO_2_ increased to 92%. The seizure activity was terminated by intravenous administration of 0.2 mg/kg of midazolam and 2 μg/kg of fentanyl. Her vital signs upon seizure termination were as follows: Glasgow Coma Scale (GCS) of 3 (eyes 1, verbal 1, motor 1), pupils 3 mm in diameter and reactive to light stimuli bilaterally, body temperature 38.2 °C, heart rate 185 beats per minute (bpm), systolic blood pressure 107 mmHg, diastolic blood pressure 60 mmHg, and SpO_2_ 99% under BVM ventilation with 100% oxygen supplementation. The patient remained apneic, and SpO_2_ quickly dropped to 50% when BVM ventilation was temporally interrupted. Venous blood gas analysis revealed respiratory and metabolic acidosis. An endotracheal tube of 4 mm in diameter was inserted for airway protection. Copious pink frothy airway secretions were identified upon intubation, and the discharge continued until the application of mechanical ventilation with positive end-expiratory pressure (PEEP) of 6 cmH_2_O. SpO_2_ was maintained above 95% after introduction of mechanical ventilation. There were no apparent injuries or hemorrhage in the oral and nasopharyngeal cavities, nor was there laryngeal edema or airway obstruction.

**Fig. 1:**
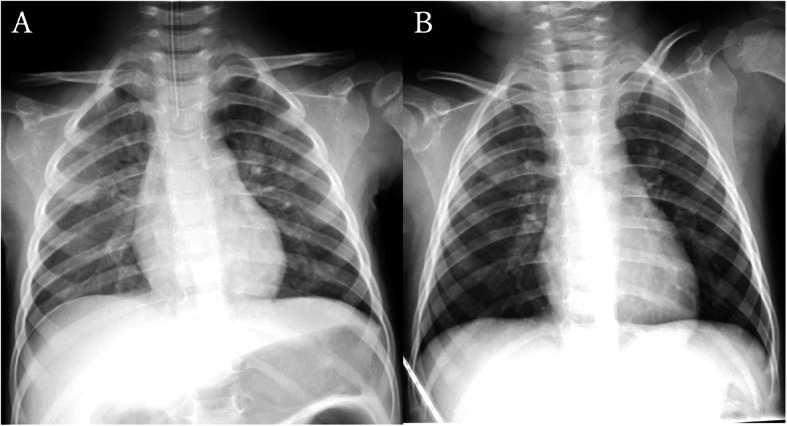
Chest radiograph findings. **a**. Revealing bilateral, centrally distributed opacities, without cardiac dilation upon ER arrival. **b**. Remarkable improvement of lung consolidation can be observed on hospital day 2

Chest auscultation revealed bilateral coarse crackles, but no heart murmur was detected. An abdominal examination revealed no hepatomegaly or splenomegaly. Chest radiography showed bilateral, centrally distributed opacities without cardiac dilation (Fig. 1a). Echocardiography demonstrated normal cardiac wall motion with ejection fraction of 69% and non-pathological trivial tricuspid regurgitation. An electrocardiogram revealed sinus tachycardia of 151 bpm without significant ST-T changes, atrial overload, or ventricular hypertrophy. Laboratory examinations revealed a mildly elevated white blood cell count, hyperglycemia, hyperammonemia, acidemia, hypercapnia, and lactic acidosis (Table 1). Creatine kinase (CK), platelet count and coagulation times were within normal limits. Computed tomography (CT) of the head revealed no abnormal findings, and continuous electroencephalography (EEG) findings did not suggest encephalopathy. A cerebrospinal fluid examination revealed no elevation in cell counts. Rapid immunochromatographic tests of nasopharyngeal specimen for influenza A and B viruses were negative.

**Table 1:** Laboratory data upon admission

Blood test (venous)	Results	Reference
White blood cells (/μL)	13,300	4000–10,000
Neutrophil (%)	43	
Lymphocyte (%)	46	
Monocyte (%)	10	
Eosinophil (%)	1	
CRP (mg/dL)	0.11	< 0.5
AST^a^ (U/L)	76	10–40
ALT (U/L)	25	5–45
LDH^a^ (U/L)	516	120–240
Total bilirubin (mg/dL)	0.3	0.2–1.2
NH_3_ (μg/dL)	124	30–86
Creatinine kinase (U/L)	131	40–150
Ferritin (ng/mL)	62.5	9.0–275
Glucose (mg/dL)	313	70–109
Lactate (mmol/L)	3.7	0.56–1.39
pH	6.902	7.35–7.45
PaCO_2_ (mmHg)	137.0	35.0–45.0
HCO_3_^−^ (mmol/L)	25,6	20–26
Base excess (mmol/L)	−12.7	−3.3 - 2.3

^a^ Hemolytic by blood sampling technique

*Abbreviations*: *CRP* C-reactive protein, *AST* aspartate transaminase, *ALT* alanine transaminase, *LDH* lactate dehydrogenase

Hypercapnia resolved after introduction of mechanical ventilation, and SpO_2_ was maintained above 95% with PEEP of 6 cmH_2_O and FiO_2_ of 0.3 or less. Pink frothy airway secretions persisted for 6 h after endotracheal intubation and then spontaneously resolved. Intravenous administration of 300 mg/kg/day of ampicillin/sulbactam (ABPC/SBT) and 20mf/kg/day of acyclovir (ACV) were empirically initiated. The patient was managed under continuous infusion of midazolam and EEG monitoring. There was no recurrence of seizure activity, and the patient became responsive without apparent neurological deficit nine hours after admission. The midazolam infusion was terminated, and the patient was extubated uneventfully 10 h after admission. There was no sign of respiratory distress after extubation, and the oxygen therapy was terminated when improvement of chest radiograph findings was confirmed on hospital day 2 (Fig. 1b). Blood, cerebrospinal fluid, and urine cultures were all negative for bacteria. Tracheal secretion cultures were positive for methicillin-susceptible *Staphylococcus aureus*, although the patient revealed no sign of bacterial pneumonia. Real-time PCR analysis did not reveal herpes simplex virus in cerebrospinal fluid, or human herpesviruses 6 and 7 in serum. ABPC/SBT and ACV were terminated on hospital day 6, and the patient was discharged. The follow-up EEG examination at one month after discharge was normal and the patient remained free of complications.

Molecular biological diagnostic assays were conducted for comprehensive detection of viral pathogens from the patient’s nasopharyngeal specimens collected upon admission and on hospital day 2. The BIOFIRE FILMARRAY Respiratory Panel (bioMérieux, France) and real-time RT-PCR were used to target predefined respiratory pathogens, and metagenomic RNA-Seq using NGS was performed to detect pathogens in unbiased fashion (Table 2) [24–30]. The BIOFIRE FILMARRAY Respiratory Panel (BioMérieux, France) does not targeting influenza C virus (FluC), however, it detected human coronavirus HKU1 (hCoV-HKU1) and human parainfluenza virus 2 (HPIV2) from the initial specimen. Real-time RT-PCR is an assay targeting 19 respiratory viruses including FluC. It identified hCoV-HKU1, FluC, and HPIV2 with quantification cycles (Cq) values of 21.5, 19.7, and 36.4, respectively. The following respiratory pathogens were not detected by BIOFIRE FILMARRAY Respiratory Panel or by real-time RT-PCR assay; influenza A (subtype H1pdm and H3), influenza B, human metapneumovirus, human respiratory syncytial viruses A and B, human bocavirus, rhinovirus, human respiroviruses 1 and 3, human parainfluenza virus 4, human adenoviruses (including 2 and 4), human coronaviruses NL63, OC43, and 229E, *Bordetella pertussis*, *Chlamydophila pneumoniae*, and *Mycoplasma pneumoniae*. NGS revealed hCoV-HKU1 and FluC, but HPIV2 was not identified. A nasopharyngeal specimen collected on hospital day 2 was analyzed by real-time RT-PCR, revealing hCoV-HKU1 and FluC with Cq values of 30.0 and 25.9, respectively, but HPIV2 was not detected.

**Table 2:** Findings of three types of genetic test assays of nasopharyngeal specimen upon admission

	Real-time RT-PCR^a^ (Cq value)	BIOFIRE FILMARRAY Respiratory Panel^b^	Metagenomic analysis using NGS; number of reads (Total, 17,937,281 reads obtained as RNA-Seq metagenomics)
Nasopharyngeal specimen Upon admission	Human coronavirus HKU1 (21.5) Influenza C virus (19.7) Human parainfluenza virus 2 (36.4) (16 viruses undetectable)	Human coronavirus HKU1 + Human parainfluenza virus 2 + (12 viruses and 3 bacteria undetectable)	Human coronavirus HKU1 714 reads (0.004%) Influenza C virus 4698 reads (0.026%) Human 16,716,897 reads (93.1%) (No other viruses read)
Nasopharyngeal specimen Hospital day 2	Human coronavirus HKU1 (30.0) Influenza C virus (25.9) (17 viruses undetectable)	NE	NE
Serum on admission	NE	NE	No viruses read
Cerebrospinal fluid upon admission	NE	NE	No viruses read

^a^ Targeting nineteen respiratory viruses: influenza A (subtype H1pdm and H3), influenza B and C, human metapneumovirus, human respiratory syncytial viruses A and B, human bocavirus, rhinovirus, human respirovirus 1 and 3, human parainfluenza viruses 2 and 4, human adenoviruses 2 and 4, human coronaviruses NL63, OC43, HKU1 and 229E

^b^ Targeting fourteen respiratory viruses: influenza A and B, human metapneumovirus, human respiratory syncytial virus, rhinovirus/enterovirus, human parainfluenza viruses 2 and 4, human adenoviruses 2 and 4, human adenovirus, human coronaviruses NL63, OC43, HKU1 and 229E. Targeted bacteria: *Bordetella pertussis*, *Chlamydophila pneumoniae*, *Mycoplasma pneumoniae*. Influenza C virus was not included in the targeted panel and was thus undetectable

*NE* not examined. +, positive

## Discussion and conclusions

We report the pediatric NPE case following febrile SE, in whom three respiratory viruses were co-detected from the patient’s nasopharyngeal specimens using molecular biological diagnostic methods. NPE following febrile SE in children is a rare but serious life-threatening complication [11–13]. Despite the higher incidence rate of FS in Japanese children than in children in other countries, few Japanese patients with NPE caused by febrile SE have been reported in literature [31–33].

NPE was suspected to be the etiology of our patient’s acute respiratory failure. Pulmonary edema was diagnosed by pink frothy airway secretions. There was prolonged oxygenation failure, even after resolution of the SE-induced apnea, with bilateral coarse crackles upon auscultation, and characteristic chest radiograph findings.

NPE is essentially a diagnosis of exclusion. The echocardiography revealed intact cardiac function, and electrocardiogram and CK measurement revealed no evidence of myocardial injury. Intravenous volume resuscitation was not conducted prior to the presentation of pulmonary edema. These findings rule out the possibility of cardiogenic pulmonary edema. The absence of airway obstruction upon intubation makes the negative pressure pulmonary edema an unlikely etiology. Viral, bacterial, and aspiration pneumoniae, as well as acute respiratory distress syndrome, do not fit the clinical presentation of respiratory failure with rapid development and resolution. The absence of hemorrhagic injury in the oral and nasopharyngeal cavities excludes the possibility of blood aspiration.

Our case has some limitations regarding the diagnosis of NPE. Arterial blood gas measurement was not conducted because of the avoidance of arterial catheter insertion in pediatric patients in our facility. This prevented us from evaluating respiratory function indices such as PaO_2_/FiO_2_ ratio and alveolar-arterial gradient. The more specific markers of myocardial injury and volume overload were not measured, including creatine kinase-muscle/brain, cardiac troponin, and brain natriuretic peptide. Despite these limitations, exclusion of other common etiologies led us to assume that NPE following SE was the most likely etiology of our patient’s acute respiratory failure.

The pathophysiology of NPE remains unclear; however, a massive increase in sympathetic activity following CNS insult is thought to be the major initial factor [1–3]. The hypothalamus and medulla are thought to be the anatomical origins of NPE, and the identified triggers of NPE include enterovirus-71-associated brainstem encephalitis, subarachnoid hemorrhage, traumatic brain injury, and epilepsy with generalized seizures or SE [2, 34]. It is hypothesized that sympathetic overstimulation leads to generalized vasoconstriction, resulting in pulmonary hypertension and increased pulmonary capillary hydrostatic pressure [35]. The increased permeability of pulmonary capillaries is suspected to be another mechanism of NPE, which can be attributed to the stimulation of alpha- or beta-adrenergic receptors by sympathetic overactivity, as well as to the release of cytokines by triggering cerebral lesions [1, 2, 35, 36]. The management of NPE includes rapid cessation or alleviation of CNS insult and supportive treatment of pulmonary edema. In our case, febrile SE was rapidly terminated upon hospital arrival, and oxygenation was secured by introduction of positive pressure mechanical ventilation. Timely achievement of both resulted in the favorable outcome of our patient.

Three respiratory viruses were identified from our patient’s nasopharyngeal specimens; hCoV-HKU1, FluC, and HPIV2. HCoV-HKU1 mainly causes upper respiratory tract infection, and higher incidence of FS complication is reported compared to other respiratory viruses [37, 38]. Acute upper respiratory tract infection caused by FluC is often accompanied by mild symptoms; however, there are reports of severe pneumonia and acute encephalopathy in children [39–41]. HPIV2 causes lower respiratory tract infection, and it is responsible for the majority of viral croup cases together with HPIV type 1 [42–44].

Our findings regarding pathogens using molecular diagnostic methods requires cautious interpretation. Genomic detection by molecular biological diagnostic methods do not certify the viability or pathogenicity of detected pathogens [18, 19]. It can be difficult to distinguish whether the infection of detected pathogen is symptomatic or asymptomatic, especially when several pathogens are co-detected, even from healthy, asymptomatic individuals [14, 18]. The viral loads obtained from real-time RT-PCR assay can be influenced by individual clinical specimens’ collection methods. Viral load standardization by reference genes in clinical specimens is not established, prohibiting comparing viral loads among various clinical samples. It is proposed, however, that pathogens with high viral loads are likely to be significantly associated with respiratory infections [45]. The real-time RT-PCR analysis upon consecutively collected clinical specimens during symptomatic periods, or upon samples from both symptomatic and asymptomatic periods, might help elucidate the clinical significance of detected pathogens.

The clinical relevance of the three detected viruses in our patient remains speculative. The finding of high viral loads of hCoV-HKU1 and FluC in the initial specimen upon admission suggests that hCoV-HKU1 and FluC were associated with the patient’s clinical presentation. The initial low viral load and the later disappearance of HPIV2 might have resulted from either HPIV2 infection prior to the other two viruses or asymptomatic colonization in the patient’s nasopharyngeal cavity. Considering the absence of nursey school attendance, the three respiratory viruses might have been transmitted to the patient from her older siblings. Symptomatically, it is also assumed that hCoV-HKU1 and FluC were associated with the pathogenesis of our patient’s case. Although human coronavirus is associated with various clinical presentations including acute bronchiolitis and gastroenteritis, its etiological role is most likely in pediatric FS cases [46]. HCoV-HKU1 is significantly associated with pediatric FS cases, even among human coronaviruses, which makes hCoV-HKU1 most likely responsible for our patient’s FS and NPE following the CNS insult [38]. There remains a possibility of the contribution of FluC to our patient’s clinical presentation, considering a case report of acute encephalopathy with FluC infection [41].

Interaction of multiple viral pathogens and their association with clinical symptoms remain controversial. Both negative and positive correlations between virus co-detection and clinical symptoms have been reported [20–23]. Tanner et al. suggested that the co-detection of different respiratory viruses is not random but reciprocal, either positively or negatively [17]. Regarding human coronavirus, the presence of viral co-detection was associated with an increased likelihood of lower respiratory tract disease compared to the detection of coronavirus alone [20]. This suggests the possibility that human coronavirus might demonstrate higher pathogenicity when co-existing with other viral pathogens. It cannot be determined, however, whether interaction among co-detected three respiratory pathogens contributed to the rare and severe clinical presentation of our patient.

We described a pediatric case of NPE following febrile status epilepticus. The timely termination of seizure activity and the rapid introduction of positive pressure mechanical ventilation resulted in the patient’s favorable outcome. Three respiratory viruses were identified using molecular biological diagnostic methods. Nevertheless, the clinical significance of these findings remains speculative.

## Abbreviations

ABPC/SBT: Ampicillin/sulbactam
ACV: Acyclovir
BVM: Bag valve mask
CT: Computed tomography
EEG: Electroencephalogram
ER: Emergency room
hCoV-HKU1: Human coronavirus HKU1
HPIV2: Human parainfluenza virus 2
MSSA: Methicillin-susceptible *Staphylococcus aureus*
NPE: Neurogenic pulmonary edema
PCR: Polymerase chain reaction
PEEP: Positive end-expiratory pressure (PEEP)
PICU: Pediatric intensive care unit
RT-PCR: Reverse transcription polymerase chain reaction
SE: Status epilepticus
